# Chitosan Based Materials in Cosmetic Applications: A Review

**DOI:** 10.3390/molecules28041817

**Published:** 2023-02-15

**Authors:** Karolina Kulka, Alina Sionkowska

**Affiliations:** Department of Biomaterials and Cosmetic Chemistry, Faculty of Chemistry, Nicolaus Copernicus University in Torun, Gagarin 7 Street, 87-100 Torun, Poland

**Keywords:** chitosan, cosmetics, skin, polysaccharides

## Abstract

This review provides a report on the properties and recent advances in the application of chitosan and chitosan-based materials in cosmetics. Chitosan is a polysaccharide that can be obtained from chitin via the deacetylation process. Chitin most commonly is extracted from cell walls in fungi and the exoskeletons of arthropods, such as crustaceans and insects. Chitosan has attracted significant academic interest, as well as the attention of the cosmetic industry, due to its interesting properties, which include being a natural humectant and moisturizer for the skin and a rheology modifier. This review paper covers the structure of chitosan, the sources of chitosan used in the cosmetic industry, and the role played by this polysaccharide in cosmetics. Future aspects regarding applications of chitosan-based materials in cosmetics are also mentioned.

## 1. Introduction

Natural polymers are becoming more and more popular, especially in fields such as medicine, cosmetics, food, packaging, and pharmaceuticals. This is mainly due to their biocompatibility, which is important when the material is in contact with the human body or food [1]. In addition to this feature, biopolymers meet a significant ecological requirement: thanks to their biodegradability, they do not pollute the environment. An equally important factor influencing the great interest in biopolymers is their compliance with the zero-waste aspect. Polysaccharides are one of the most commonly used groups of natural polymers in the above-mentioned areas. This group includes chitin, the second most widespread polysaccharide in nature, and chitosan, which is a chitin derivative [2].

Chitosan, in addition to the properties characteristic of biopolymers (biocompatibility, biodegradability, and non-toxicity) has a number of unique attributes. The first essential feature is its cationic character in solution. Other valuable properties include film-forming, antimicrobial, and antioxidant capacity; susceptibility to modification due to functional groups; and adsorption capacity [3,4,5]. This biopolymer can be processed in various forms—among others, powder, fiber, gel, membrane, and granules [6]. The form in which chitosan occurs is closely related to its use. Granules are often used as a biosorbent in water purification from heavy metals [7,8]. Regarding chitosan films or membranes, they are applied as active dressings, drug carriers [9], and more and more often as a base for cosmetic masks [10]. Chitosan applications in cosmetics is a relatively recent issue, but more and more scientist are taking up this topic. A limitation in its use in cosmetics and many others fields is its insolubility in water. Despite this, there are more and more formulations containing chitosan on the cosmetic market [11].

The purpose of this review is to present crucial information about chitosan and its structure and properties, and broad discussion of its current and possible applications, in particular, in the field of biomedicine and cosmetology.

## 2. The Structure of Chitin and Chitosan

**Figure 1:**
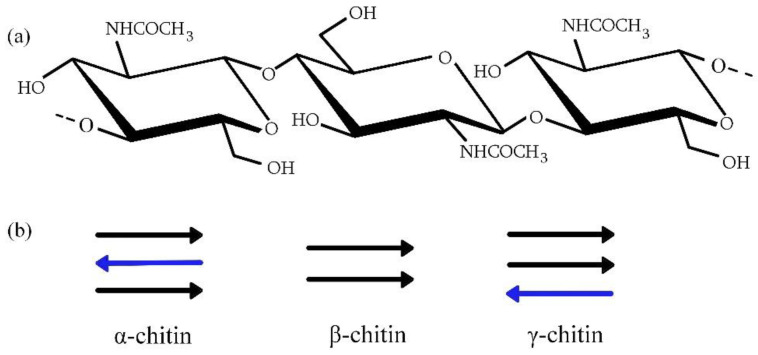
(**a**) Chitin structure; (**b**) Chitin forms (Arrows represent the conformation of the polymer strand; black arrows show the parallel arrangement of chains; blue arrows show antiparallel arrangement of chains.).

Chitin and its derivative, chitosan, are linear polysaccharides. Chitin is a building material of crustaceans’ and insects’ exoskeletons, and it is found in fungi cell walls. Mushrooms were the first historical source from which chitin was isolated [12]. Subsequently, this polymer was also found in insects, and now the most common source of this biopolymer is shrimp-processing waste [13]. The name ‘chitin’ comes from the Greek word ‘chiton’ and means tunic. Chitin is structurally related to the most widespread natural polysaccharide—cellulose; it differs from this biopolymer by the presence of the acetamide group in the C2 position. Chitin consists of N-acetyl-D-glucosamine units that are rotated 180° with respect to each other (Figure 1a). It occurs in three allomorphous forms: α, β, and γ (Figure 1b). Each chitin type differs in the chain arrangement in the crystalline region [14]. This translates into properties of the polymer—among others, the degree of hydration and mechanical properties [15]. Antiparallel conformation of the strand is characteristic of α-chitin, and parallel arrangement is typical of the β-form. The third form consists of two parallel and one antiparallel arranged chains. Crystalline modification depends on chitin origin. The antiparallel chains’ conformation in α-chitin makes its structure more stable, which is why crustaceans and arthropods are the main sources of α-chitin; but it also occurs in fungi, and the β-form is found in mollusks, such as squid, and the γ-chitin source can be insects [15,16].

**Figure 2:**
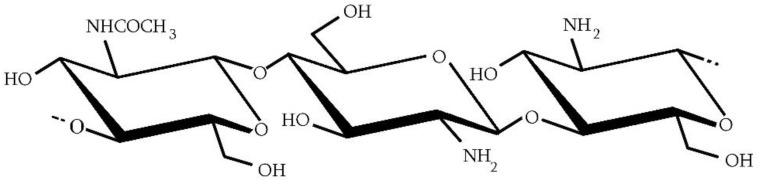
General structure of chitosan.

Chitosan is a copolymer made of two structural units: N-acetyl-D-glucosamine and D-glucosamine (Figure 2). Chitin differs from chitosan in the degree of deacetylation (DDA). DDA is the ratio of the number of D-glucosamine units to the total number of units in the polymer. If the value of this index is above 50 mol%, the product can be called chitosan. To sum up, in the polymer chain, there are units containing an amino (-NH_2_) or acetamide (-NHCOCH_3_) group in the C-2 position, and they are distributed differently. Chitosan with an appropriate DDA can be obtained as a result of chitin deacetylation or in the process of reacetylation of chitosan with other parameters. The method of obtaining chitosan may affect its structure and the arrangement of units along the polymer chain, which may lead to the creation of random-type or block-type copolymers with different properties [17]. The mentioned earlier limit value of DDA is also important because then the polymer can dissolve in dilute acid solutions, such as acetic, citric, lactic, succinic, formic, and many others [18]. The dissolution of chitosan in dilute solutions of carboxylic acids can be considered a traditional method. It is possible to dissolve chitosan in water saturated with CO_2_ under appropriate pressure [19,20]. This approach allows obtaining even better solubility results compared with dissolving in traditional solvent, of which the most commonly used is acetic acid. In this case, chitosan solubility kinetics depend on the temperature, particle size, and used pressure [20,21]. Chitosan solubility is determined by the pH of the solution, which is related to the pK_a_ value (about 6,5) [22]. Above pH = 7, the polymer cannot be dissolved because of its compact structure, that is, the high number of hydrogen bonds that can be created with the participation of -NH_2_ and -OH groups. In a slightly acidic solution below the pK_a_ value, amino groups (-NH_2_) are protonated, causing electrostatic repulsion and swelling of the polymer. Chitosan is, therefore, a cationic polyelectrolyte, the only alkaline polysaccharide observed in nature [23,24]. Thanks to this, chitosan is able to react with polyanionic compounds, forming polyelectrolyte complexes [25,26].

## 3. Sources of Chitin for Chitosan Preparation for Cosmetic Use

**Figure 3:**
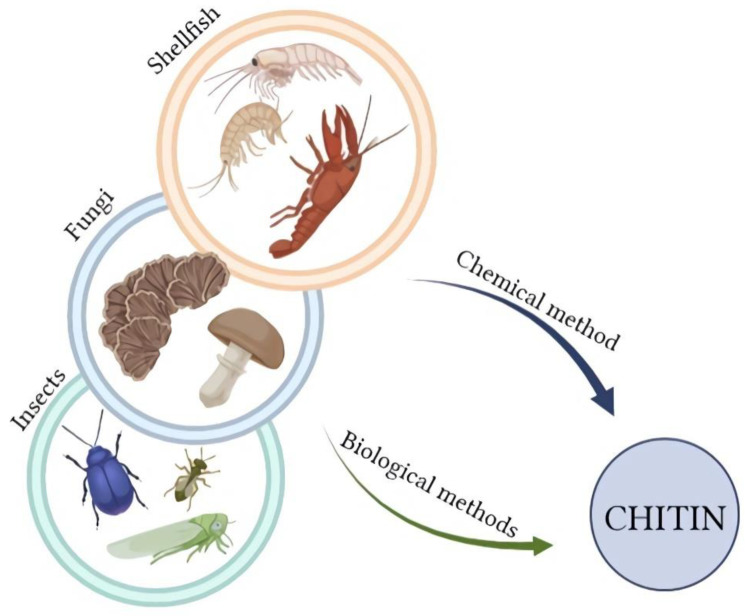
The sources of chitin for chitosan preparation (Created with BioRender.com; accessed on 4 January 2023).

Chitin is a biopolymer; therefore, the source of its acquisition is natural and renewable. The main material for chitosan production is usually waste from the biomass of marine organisms (Figure 3); millions of tons of this biopolymer are extracted annually [27]. An alternative source of chitin is insects and fungi, which have several advantages compared to crustaceans. The availability of insects is not seasonal, as well as fungi; additionally, fungi do not require a demineralization process [28]. In addition, chitin from some insect species is more susceptible to chitinase activity [16]. Examples of insects used to produce chitin are blowfly, beetle, cicada slough, or bumblebee [16]. Among the species of fungi that are the source of chitin, *Pleurotus sajor-caju*, *Lentinula edodes*, *Agaricus bisporus*, *Auricula judae*, *Trametes versicolor*, *Armillaria mellea*, and *Pleurotus ostreatus* can be mentioned [29]. There are two main ways to prepare chitin: biological and chemical [29,30]. Several stages of obtaining chitosan can be distinguished in both methods, such as the already mentioned demineralization process. Raw material contains not only chitin, but also proteins or minerals; therefore, the first stage in the preparation of this material is its drying and fragmentation, followed by deproteinization, demineralization, and decolorization [31]. Deproteinization is the first step, which requires the use of alkali and high temperature in the chemical approach, or the use of proteolytic enzymes in the biotechnological method. The main enzymes used for this purpose are: Papain, Trypsin, Chemotrypsin, Pepsin, and Pancretin [27]. During hydrolysis, enzymes cleave protein peptides, breaking chitin-protein complexes and leading to the creation of hydrolyzed protein, which remains in the soluble fraction [30]. The next stage in the chemical process is demineralization; it uses dilute hydrochloric acid [27]. In the biotechnological method, the order of these two steps is reversed. An alternative promising method of enzymatic deproteinization is fermentation, with or without lactic acid bacteria, which can be conducted by adding selected strains of microorganisms [30,32]. This innovative method is more environmentally friendly because it does not use such amounts of concentrated chemicals, and additionally, proteins obtained during enzymatic processes can be used for consumption purposes [32]. Decolorization is an optional treatment; it depends on the marine source, e.g., squid pens do not require this stage, but some crabs shells or shrimps have characteristic pink color. The last and significant step in obtaining chitosan is the chitin deacetylation process, which can be carried out by an enzymatic or chemical method. The degree of deacetylation and the average molecular weight of the final product can be controlled by varying the NaOH concentration and temperature. The disadvantages of this process are the uncontrolled hydrolysis of the polymer and the generation of large amounts of wastewater containing concentrated bases. Enzymatic deacetylation of chitin is more environmentally friendly and allows greater control of the parameters of the final product. The enzyme used for this purpose is deacetylase (EC 3.5.1.41). The products of enzymatic deacetylation of chitin are chitosan and acetic acid. Acetic acid is a deacetylation inhibitor; therefore, it is important to remove it efficiently from the reaction environment [33]. In the case of marine material, various factors, including seasonality, freshness of the raw material, quality of the shell, organism species, or even the distance to cover the delivery to the destination, affect the quality of the obtained biopolymer, and thus its properties. Chitosan can have completely different physical characteristics: color, density, and particle size. Years of research on the production of chitin and chitosan from aquatic organisms allowed for the improvement and unification of the entire process.

## 4. Chitosan Applications

**Figure 4:**
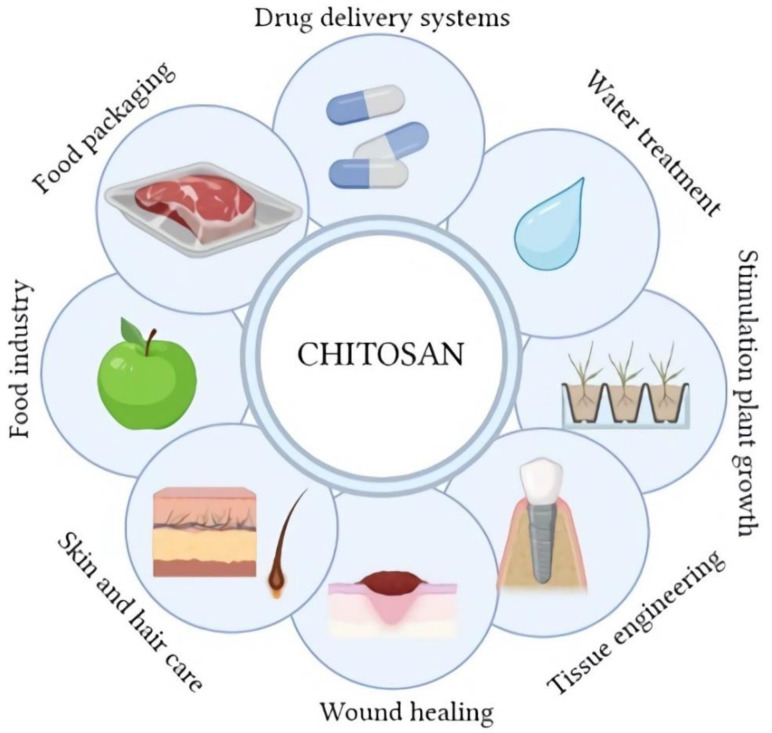
The main applications of chitosan (Created with BioRender.com; accessed on 4 January 2023).

Chitosan as a biopolymer is of great interest, especially in areas where safety is essential, such as many branches of biomedicine, pharmacy, cosmetics, and the food and food packaging industries (Figure 4). In each of these cases, the potential use of chitosan is strictly connected with the human body [1]. Chitosan is characterized by biocompatibility and non-toxicity, which are crucial in the above-mentioned applications. An additional attribute of chitosan is a number of specific properties and biological activity that largely determine the multitude of its applications. Antioxidant activity is one of the most frequently mentioned abilities of this polymer [18,34,35,36,37]. There is a correlation between the level of this activity and some parameters of chitosan. A polymer with a higher degree of deacetylation and a greater number of unsubstituted amino groups exhibits increased antioxidant activity. High average molecular weight reduces this activity, which is explained by the presence of more external hydrogen bonds [34,35,38]. The food packaging and food industries use the above-mentioned activity of chitosan to produce active and edible films [39,40,41]. A complimentary, if not more important, aspect of this type of chitosan application is the proven antimicrobial activity [40,42,43,44,45]. Modern food chitosan packaging can be divided into active release systems and active scavenging systems [41]. In both types of packaging, chitosan is often used as the main matrix, to which other additives are added to enhance the antioxidant and antimicrobial effect. The additives used in such packaging are often substances of natural origin, such as plant extracts or essential oils [46,47,48,49,50,51,52]. Currently, the most commonly used solution in securing food is the surface application of chitosan [42,45]. The antibacterial activity of chitosan is due to its polycationic character. Protonated amino groups bind negatively charged groups of LPS lipopolysaccharides and peptidoglycans on the surface of pathogen cells, which results in the destruction of their membranes [38,45]. The higher molecular weight of chitosan reduces this activity because it is unable to penetrate the cell walls of microorganisms. However, this parameter does not completely inactivate the antimicrobial activity of the polymer, which is able to chelate metal ions and change the permeability of the pathogen cell wall, limiting the exchange of nutrients [39]. The level of chitosan antimicrobial activity also depends on the type of pathogen; the greater the negative charge on the cell surface, the stronger the effect [39,42]. It is also possible to influence chitosan oligomers on intracellular structures, such as genetic material, and influence protein synthesis pathways [39,53]. The main tasks of this type of packaging is to ensure consumer safety and increase the shelf-life of food products [41].

Biomedical applications of chitosan are broad and diverse. In addition to the already mentioned antioxidant and antimicrobial effects, chitosan accelerates wound healing, has great mucoadhesive characteristics and film-forming and anti-inflammatory properties [54,55]. Chitosan-based products are mainly used in wound-healing, tissue-engineering, and drug-delivery systems [56,57,58,59]. The wound-healing process involves several steps, e.g., inflammation, migration, proliferation, and maturation, ending with remodeling [60,61]. The course of the regenerative process depends on many factors, such as the age of the wound, the thickness of the wound, the origin of the injury, and its complexity [61,62]. Chitosan-based formulations supporting the wound-healing process can come in various forms, such as gel, sponge, or active dressing [60,63,64]. Chitosan is a basis for such products, to which are added other active substances, such as medicines. This combination gives a synergistic, positive healing effect, and then chitosan serves two functions—an active dressing and a drug carrier [64]. The mechanism of chitosan action for the healing process is, again, related to its polycationic nature, which enables its molecules to bond with negatively charged thrombocytes and erythrocytes, which facilitates the blood-coagulation process. In the case of the active dressing form, an additional function is the formation of the occlusal layer, which maintains the proper moisture level and the ability to absorb exudate from the wound [60,62,64,65].

Another area of extensive use of chitosan is drug delivery. There are several ways to deliver drugs, including the oral route, ocular route, application to the skin, etc.; it depends on the medical need. Oral drug delivery with the usage of chitosan gives promising results, especially in terms of drug release depending of the pH, which is particularly important for the release site [66,67,68]. Chitosan can be administered orally alone for therapeutic purposes, too. It has been proven that it lowers cholesterol and triglyceride levels and reduces the risk of cardiovascular diseases [38,58]. Low-molecular-weight chitosan (LMWC) is used in this case. The therapeutic effect is probably due to the binding of anionic fatty components, such as fatty acids and bile acids, to the positively charged chitosan. In parallel, low-molecular-weight chitosan consumed with fats can trap fat molecules in the stomach, and when this system reaches the small intestine, along with the change in pH, it precipitates and prevents the absorption of fats [58]. Chitosan is also considered as a brain drug delivery carrier in the treatment of diseases, such as cancer, epilepsy, Alzheimer, Parkinson, or migraine [57]. Once again, the main advantage of chitosan is its polycationic nature, thanks to which it can be absorbed by negatively charged cell membranes and support the penetration of drugs through the blood–brain barrier [57]. Chitosan or its modifications were used in many studies in this field, e.g., derivatives (carboxymethyl chitosan) or nanostructures (nanocapsules, nanoparticles, micelles, and nanoemulsions) [69,70,71,72,73,74]. Different administration routes of this type of therapeutic have been used, of which the nasal route is the most common, which allows for obtaining a sufficiently high concentration of the drug in the brain compartment [57,70,71]. Other routes of brain drug delivery include oral and intravenous application [57].

In recent years, there has been a strong trend in the use of biomaterials, including chitosan, in tissue engineering and regenerative medicine. Tissue engineering uses knowledge and achievements from other fields, including biology, medicine, and nanotechnology [75]. A number of requirements are placed on the materials used in this interdisciplinary field. First of all, the material should be biodegradable, have appropriate mechanical properties so that it can imitate the replaced tissue, be able to be properly formed, and should promote the attachment of cells and their differentiation and proliferation [75,76]. Chitosan is a suitable candidate for the application as a scaffold matrix, as it fulfills the above conditions. The main areas of application of tissue engineering include the regeneration of skin, bone, neural tissue, cartilage, and dentistry [77,78,79,80,81,82,83,84]. Scaffolds can take different forms. Depending on the application, the main types can be distinguished: porous, fibrous, hydrogel, microsphere, composite, or acellular [77]. Skin injuries can be caused by a variety of factors and can affect different areas. Dangerous for health and life are large areas of damaged skin. Auto and allogeneic grafts give good results, but they carry some risks, including those related to surgery, infections, or scar formation [75,77]. Skin tissue engineering offers scaffolds that are a combination of biomaterials, including chitosan. These materials are often loaded with growth factors, antibiotics, and other supporting substances [77]. There are many references to the use of chitosan-based hydrogels as 3D scaffolds, and the main method of forming such structures is electrospinning [75,84,85]. Fischetti et al. investigated a chitosan and gelatin blend with tripolyphosphate as a crosslinking agent for suitability as bio-ink for 3D printing to be used as scaffolding [86]. The results of the study showed that the proposed material is compatible with cells in the in vitro test, and has good stability and a slightly lower modulus of elasticity than native tissue, which, however, is not a factor that excludes the use of this composition. In bone tissue engineering, the mechanical parameters of the scaffold used are important and should be, preferably, as close as possible to bone tissue. Chitosan alone does not meet these conditions, but it is often combined with hydroxyapatite or simply coated on implants [87,88,89]. In the case of nervous tissue, it does not have such a high regenerative capacity as the discussed tissues, especially when it comes to the axons of the central nervous system. Currently, tissue engineering based on biomaterials gives positive effects related to the differentiation of neural stem cells or the growth of neurites [80].

The applications of chitosan presented above are only a fragment of the possible ways of using this biopolymer. Other applications include, among others, purification of water from heavy metals and sorption of dyes, reducing the turbidity of water, production of contact lenses, and stimulation plants growth [90,91,92,93,94].

## 5. The Role of Chitosan in Cosmetics

Chitosan is not a very popular ingredient in cosmetics compared to other biopolymers, such as collagen or hyaluronic acid; but the interest in this topic is growing. The main factor influencing this is the limited solubility of chitosan in water. However, more and more products with chitosan are introduced in the market due to the multitude of beneficial functions it can perform in the formulation. Even if not chitosan itself, its derivatives are used for many cosmetic applications [11,95,96]. Chitosan is an ingredient approved for use in cosmetics by the FDA and the EU. In the European Union, cosmetics are subject to the regulations contained in Regulation (EC) No 1223/2009 of The European Parliament and of the council of 30 November 2009 on cosmetic products. This biopolymer is not on the list of substances not allowed or allowed with restrictions for use in cosmetics. According to the COSING database, which is part of the official website of the European Union, chitosan is assigned two cosmetic functions: film forming and hair fixing. Moreover, chitosan binds water, hydrates the skin, and can be used as a thickener, rheology modifier, and emulsion stabilizer [97,98]. It creates a hydrophilic film on the skin, preventing water loss [99]. The antimicrobial activity of chitosan has a double meaning; it is then present as an active substance and, thus, it is possible to reduce the use of preservatives in the formulation. It also has an affinity for keratin, so it is successfully used in haircare products [11]. Film-forming properties allow the use of chitosan in cosmetic masks that work on a similar principle as wound dressings. This biopolymer can be an ingredient of emulsions, gels, foams, sticks, or aerosols in every type of cosmetics—intended for use on skin, hair, or nails or in oral hygiene preparations [2,11,99].

**Figure 5:**
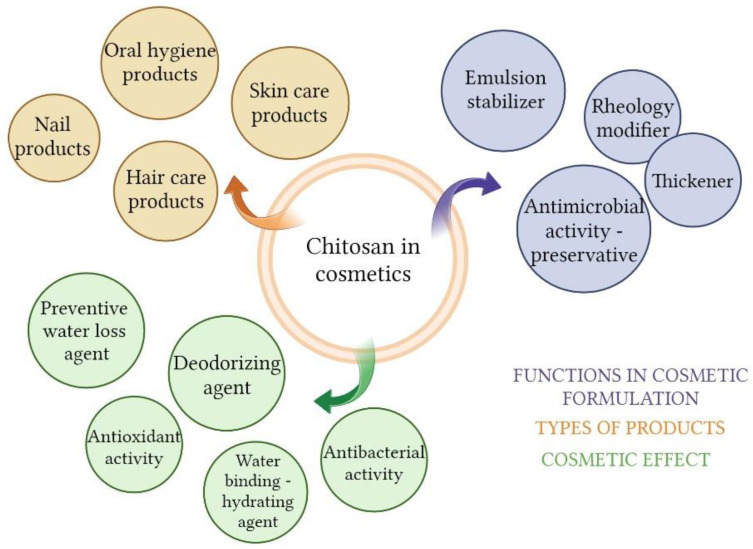
Chitosan in cosmetics (Created with BioRender.com, accessed on 9 February 2023).

In the previous section, a number of applications of chitosan in biomedicine were indicated. Many of them related to the skin or mucous membranes are an inspiration to create effective and safe cosmetics (Figure 5). The goal in cosmetics is not always to achieve an anti-aging effect. Cosmetology supports the treatment of skin diseases or helps to minimize their effects. The most common skin illnesses that need cosmetology support are acne, hyperpigmentation, depigmentation, psoriasis, acne and post-surgical treatment scars. Appearance strongly affects self-esteem, social life, quality of life, and general mental condition [100,101]. Proper care helps to keep the skin in good condition and appearance. An example of the cosmetics industry drawing inspiration from biomedical sciences is the already mentioned beauty masks similar to wound dressings, as well as the encapsulation of active ingredients using chitosan [9,102,103,104].

Thanks to its antimicrobial activity, chitosan is applicable in deodorants and antiperspirants, where it is a breeding ground for bacteria contained in sweat, which reduces the formation of odor-causing metabolites [105,106]. Thanks to these properties, it is also a desirable ingredient in anti-acne cosmetics [107,108]. Antimicrobial activity has a significant matter in oral healthcare, where bacteria play a crucial role in the development of dental plaque [11]. Chitosan is used in the production of chewing gums, toothpaste, and rinses [11,44,66] (Table 1).

The structure of the hair is complex, and its main component is keratin. The outer sheath of the hair is constantly exposed to destructive factors, including mechanical, UV light, high temperatures during modeling, or chemical factors during dyeing. Chitosan and other biopolymers improve the formulation consistency and adhering of other ingredients to hair [11]. Furthermore, the conditioning action of chitosan results from its positive charge that neutralizes the charge of damaged hair. Hair conditioning cosmetics with chitosan can fix the structure of the hair by forming a film on its surface. This action reflects on the appearance of hairs, which are softer and thicker [103]. Kojima et al. checked chitosan’s ability to penetrate into the hair with time-of-flight secondary ion mass spectrometry (TOF-SIMS). They compared dyed hair to normal hair (undyed). Results showed a higher degree of chitosan penetration in dyed hair. This indicates and confirms the destructive effect of hairdressing treatments and the ability of chitosan to incorporate to the hair structure [111].

Another potential role of chitosan in cosmetics concerns protection against UV radiation. Biopolymer shows absorption below 400 nm; therefore, it has photoprotective potential [11]. However chitosan’s gel SPF rate is very low [112]. Chitosan may be more useful in this regard as an ingredient that enhances the effectiveness of other UV filters or mitigates the effect of UV radiation on the skin. Bikiaris et al. obtained chitosan nanoparticles and carried out the encapsulation of pomegranate juice. Subsequently, they introduced the loaded nanoparticles to emulsions. Results showed enhanced UV protection in systems with chitosan compared to the control sample [113].

The use of this polysaccharide as a potential component of reusable filtering masks is also worth mentioning. Due to the coronavirus pandemic, huge amounts of face masks are needed, both among healthcare professionals and also among citizens, as a means of personal protection. Daily use of disposable protective masks is recommended, which generates huge amounts of waste. Choi et al. proposed a new solution for reusable masks with filters. They fabricated a membrane filter integrated with poly(butylene succinate)(PBS) microfiber and nanofiber mats and coated this with chitosan nanowhiskers. The tested filter effectively captured particulate matter (PM), providing a comfortable breathing environment. The proposed filter is durable, enabling its repeated use. The results of biodegradation tests showed that it decomposes in the soil after one month. It is predicted that it may have antibacterial and virus-blocking properties. It is a promising alternative to disposable filters [114].

## 6. Modification of Chitosan for Cosmetic Applications

**Figure 6:**
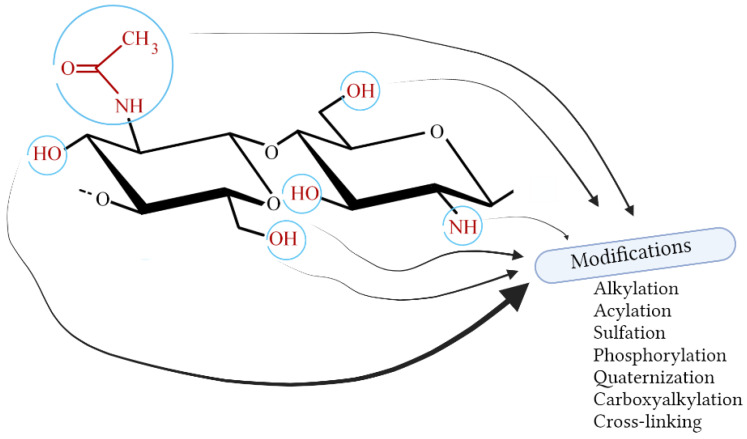
Selected modifications of chitosan (Created with BioRender.com; accessed on 9 February 2023).

**Figure 7:**
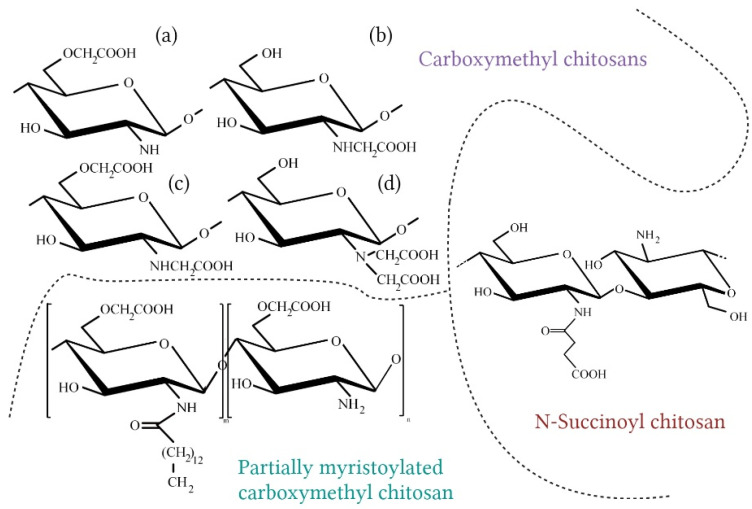
Selected chitosan derivatives for cosmetic applications (**a**) O-carboxymethyl chitosan; (**b**) N-carboxymethyl chitosan; (**c**) N,O-dicarboxymethyl chitosan; (**d**) N,N-dicarboxymethyl chitosan (Created with BioRender.com; accessed on 13 February 2023) [95,115,116,117].

The presence of functional groups in chitosan (hydroxyl, amino, and acetamide) gives a wide range of possibilities for its chemical modification (Figure 6 and Figure 7). For instance, the processes of alkylation, acylation, sulfation, quaternization, phosphorylation, and carboxyalkylation can be carried out [115]. Other modifications include oligomerization and graft copolymerization. The oligomerization process is very useful, especially in the cosmetic field; smaller molecules dissolve better and can penetrate membranes easier [116]. Oligomers of chitosan can be obtained by various methods: chemical, enzymatic, or physical. The chemical one is non-specific, and the hydrolysis goes randomly. Enzymatic oligomerization can be carried out with various enzymes, not only chitinase, and allows for greater control of the entire process. Physical methods include, among others, sonication [115].

Currently, a huge number of chitosan modifications, mainly chemical, can be distinguished. In the cosmetics field, there is a constant need for new solutions and new ingredients. According to the CoSing database (https://ec.europa.eu/growth/tools-databases/cosing/index.cfm?fuseaction=search.results, last accessed on 15 December 2022), 50 different forms of chitosan are currently used in cosmetics; this is 6 items more than in 2018 [11].

The advantage of carrying out the chitosan modification process is that the polymer skeleton is not affected, it retains its basic properties, and it is additionally enriched with a new one [115]. Some of the most significant chitosan derivatives in cosmetics are discussed below.

Carboxymethyl chitosan (CMCh) is one of the best known chitosan derivatives. It is the product of the carboxyalkylation process. It introduces acidic groups on the chitosan chain; therefore, it has an amphoteric character. Depending on the reaction conditions, different types of products can be obtained. The substitution may occur at the C-6 hydroxyl group or amino group, leading to the following forms: N-Carboxymethyl chitosan, O-Carboxymethyl chitosan, N,O-dicarboxymethyl chitosan, or N,N-dicarboxymethyl chitosan. The N-carboxymethyl form has numerous advantages—high viscosity, water-holding capacity, film and gel-forming properties, and soluble in water in neutral pH—which makes this compound a desirable cosmetic ingredient [115,117]. An important parameter characterizing CMCh is the degree of substitution (DS), which determines the solubility of the polymer [117]. Tzaneva et al. have obtained CMCh with 50% DS and introduced the polymer to emulsions. They concluded that this chitosan derivate improves rheological properties of the emulsion and can replace one of the most frequently used stabilizers, Carbomer [118]. CMCh also has antibacterial properties; it shows even stronger effects than chitosan [117]. Farag et al. have examined the antimicrobial and antifungal activity of CMCh nanogel, and the results showed the effectiveness of this compound against *Escherichia coli*, *Staphylococcus aureus*, *Aspergillus flavus*, and *Candida albicans* [119]. Carboxymethyl chitosan was also tested as a potentially supportive deodorant agent. Chaiwong et al. have examined cosmetic formulations with this polymer and mangosteen extract, and the results indicated the synergistic activity of these ingredients against trans-2-nonenal odor, which is an unsaturated aldehyde obtained from lipid oxidation that has an unpleasant smell. What is more, creams with the mentioned combination of compounds had good moisturizing, antioxidant, and antibacterial properties. The optimal concentration of CMCh for emulsion stability was also determined (it allowed maintaining the viscosity and pH during storage), and it was 1% (*w*/*v*) [106].

N-Succinoyl chitosan (NSCS) is the water soluble product of succinic anhydride and chitosan reaction. It performs skin-conditioning and protection functions. This derivative has excellent mucoadhesive properties, thanks to the presence of the carboxylate group from succinic acid [120]. Other properties include prolonged circulation time in the human organism, pH-sensitivity, and low-cytotoxicity, which make this derivative a suitable ingredient with biomedical applications [120,121]. Li et al. carried out C-6 selective oxidation of NCSC using a TEMPO/NaOCl/NaBr system. Products of this process exhibit very good water absorption and retention abilities, potentially better than hyaluronic acid, which has been one of the most commonly used biopolymers with such properties for years [121].

Partially myristoylated carboxymethyl chitosan (PMCC) is an amphoteric and amphiphilic derivative with the ability to form micelles [95]. The convenience of using PMCC compared to the starting polymer is the lack of precipitation in the presence of anionic compounds. Chitosan creates polyion complexes, with anionic polymers manifested by precipitation and the instability of cosmetic formulation, which is an undesirable phenomenon. Seino et al. examined a PMCC compound in combination with carboxyvinyl polymer and obtained very stable translucent gel. This derivative is a promising cosmetic ingredient that potentially facilitates the penetration of active ingredients through the stratum corneum [95].

**Figure 8:**
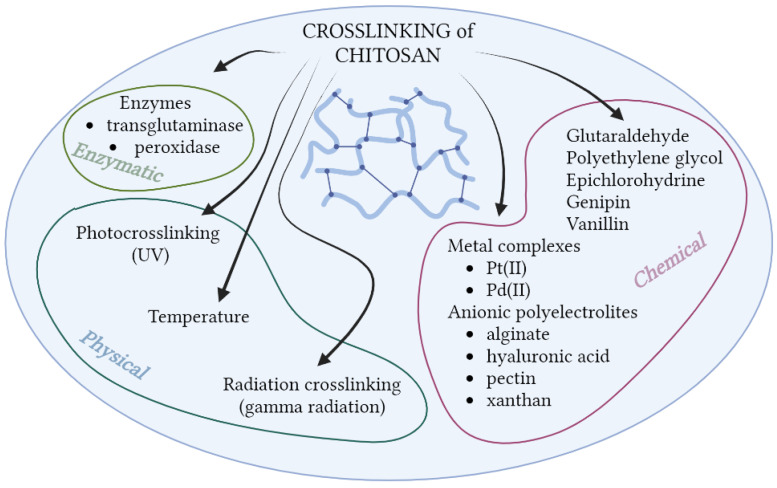
Crosslinking agents for chitosan (Created with BioRender.com; accessed on 18 January 2023).

In addition to the above-mentioned methods of chitosan modification, crosslinking is another one. It allows obtaining a three-dimensional polymer network (Figure 8). A polymer crosslinked structure is formed as a result of the formation of bonds between the polymer chains or between polymer chains and a multifunctional crosslinking agent. This effect can be achieved by the use of chemical or physical agents. Responsible for the formation of the polymer network are most of all covalent and ionic interactions, but also hydrogen bonds and hydrophobic ones. The crosslinking process changes the properties of the polymer, including increasing its mechanical resistance [122]. Crosslinking of chitosan is associated with the formation of hydrogels, which are of great interest to scientists. These systems can absorb and keep a huge amount of water, and by this way have very useful properties because it makes them similar to human tissues. Hydrogels based on chitosan are an excellent material for encapsulation, wound dressings, and the design of drug-release systems [85,105,122]. Covalent crosslinking is permanent and allows for the absorption of ingredients and their controlled release, while ion-crosslinked polymer is more susceptible to pH changes [103]. The best-known chemical crosslinking compounds of chitosan are glutaraldehyde (GA), genipin, and polyethylene glycol [123,124,125,126,127]. Currently, many new crosslinking compounds are used, mainly of natural origin, such as vanillin [128]. As in the case of the discussed modifications in the previous section, crosslinking of chitosan takes place with an amino or hydroxyl group from C-6. Ostrowka-Czubenko et al. prepared hydrogel membranes based on chitosan and glutaraldehyde (GA) or GA and sulfuric acid (SA). The results of FTIR spectroscopy confirmed the formation of covalent and ionic crosslinks between the polymer and added agents. The swelling ratio of both obtained membranes showed an increase in alkaline media [129]. Additionally, thanks to its polycationic nature, chitosan can easily react with anionic compounds, undergoing ionic crosslinking. Ionic crosslinking can occur in the presence of low-molecular factors, such as metal complexes (Pt(II), Pd(II)) [130,131], or anionic polyelectrolytes (e.g., alginate, hyaluronic acid, xanthan, and pectin) [132,133,134,135], which leads to the formation of polyelectrolyte complexes. Wang et al. prepared chitosan-alginate hydrogel for tissue-engineering applications. The FTIR spectroscopy and X-Ray diffraction results confirmed strong ionic interactions between chitosan and alginate. Prepared complex with a highly hydrophilic character, porous structure, and good cell compatibility makes it a suitable material for scaffold production, even in such sensitive areas as neural systems [136]. Other methods of chitosan modification include its radiation crosslinking, using gamma rays, or photo-crosslinking with UV radiation [137,138]. Enzymatic crosslinking is also a promising method [139]. Mun et al. took advantage of this crosslinking method by using horseradish peroxidase (HRP) and hydrogen peroxide for crosslinking chitosan and collagen. The hydrogel forming time was 5 min in this case, which makes this type of crosslinking very fast compared to other methods of obtaining hydrogels [139]. Not only do chitosan hydrogels undergo crosslinking, but it is also possible in the case of its derivatives [103]. N-succinyl chitosan is readily used in the form of a hydrogel. Bashir et al. conducted the preparation of N-succinyl chitosan and its hydrogel using glutaraldehyde as a crosslinking agent. They examined the swelling ratio of the hydrogel in different pH values. Results confirmed hydrogel pH sensitivity (low swelling ratio at acidic pH and high at neutral pH). The swelling potential was the result of a highly porous structure that helps absorb water [120]. The potential use of chitosan hydrogels in cosmetics is mainly of importance as a superficial application, in the role of a humectant and matrix for active substances.

## 7. Chitosan Blends for Cosmetic Applications

The demand for new raw materials with better functional properties is constantly growing. Creating blends of polymers is a way to obtain materials with the desired characteristics. It is a simple, cheaper, and faster solution compared to obtaining new types of polymers. Another advantage of using mixtures of macromolecular compounds is obtaining the material with the synergistic combination of properties; it allows overcoming the deficiencies of individual components, too [140]. Biopolymers are a special group of macromolecules that can be used for this purpose because of their ecological aspect. In the literature, there are two main terms: miscible and immiscible polymer mixtures [141]. The miscible blend is a general homogenous system with single-phase properties; initial materials are dissolved in each other or in the same solvent at the molecular level. The immiscible mixtures are characterized by phase heterogeneity, which results from the lack of solubility of components in each other. Partially miscible blends can also be distinguished [140]. Miscible composite materials are desirable because of their uniform performance and stable thermal and mechanical properties [140]. There are several tools to assess the polymer miscibility—among others, Fourier transform infrared spectroscopy (FTIR) and differential scanning calorimetry (DSC) [142]. The first technique allows defining specific molecular interactions in the composition. IR spectra of miscible blends show shifts, the disappearance of some bands, or the creation of new ones [142]. DSC is helpful in the measurement of glass transition temperature (Tg), which is one of the most important thermodynamic properties of amorphous polymers [140,142]. The modification of polymer-based material can be carried out by the addition of natural or synthetic polymer. One of the ways to obtain polymer blends is by mixing their solutions (in aqua or other solvents) [143]. This approach is simple, but cannot be implemented in every case because of the insolubility of some polymers in common solvents.

**Figure 9:**
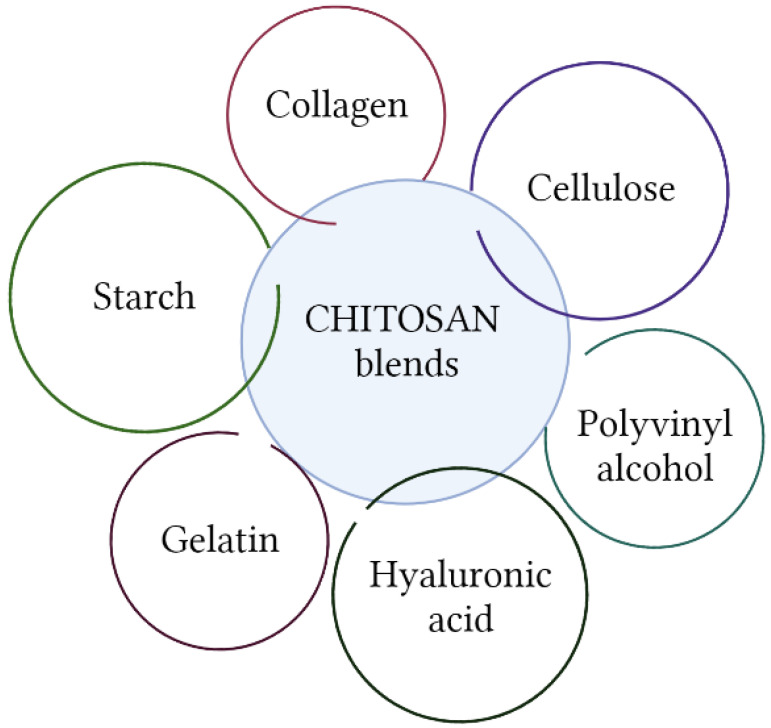
Blends of chitosan with other polymers (Created with BioRender.com; accessed on 18 January 2023).

Chitosan can be blended with other biopolymers or synthetic polymers for various purposes (Figure 9). A review of current articles indicates multiple obtainable modifications using this biopolymer. Only two-component mixtures are rarely used; modifications include the introduction of low molecular-weight additives, such as nanoparticles [144]. The aim of this process is to improve mechanical properties or enhance other abilities, e.g., antimicrobial or antioxidant. The studies discussed below are not directly related to the area of the cosmetics industry, but may find potential application in this field.

The properties of chitosan can be modified by adding polyvinyl alcohol (PVA), a synthetic polymer soluble in water. A study of the mechanical and thermal properties of this blend has been carried out by Abraham et al. [145]. Chitosan was blended with PVA at varying concentrations (2%, 4%, 6%, and 7% of chitosan) by mixing and stirring solutions of these polymers. Two modifications were also implemented by adding formaldehyde as a crosslinking agent and glycerol as a plasticizer. Thermal analysis results of obtained films pointed out a blend with formaldehyde as the most thermally stable. Mechanical test results showed that increasing the amount of chitosan decreases the mean tensile strength and percentage of elongation. Another study with the same polymers checked its application in the form of fibers [144]. More et al. additionally introduced silver and copper nanoparticles. Fibers were obtained from the prepared mixtures by the electrospinning process. A number of tests were performed to characterize the obtained structures, e.g., thermal analysis, microscopic analysis, and infrared spectra. The thermal results did not show the influence of nanoparticles on the thermal stability of the composite fibers. The tested fibers can potentially be used in wound dressings.

Collagen, as one of the most widely used cosmetic ingredients with moisturizing and anti-aging properties, would be a desirable polymer to be combined with chitosan. Chitosan/collagen blends are the subjects of many studies, mainly in the tissue-engineering field [146,147,148]. Sadeghi-Avalshahr et al. examined this composition in grafting the surface of the prepared from PCL/PVP electrospun samples. The aim of coating this scaffold with biopolymers was improving surface biocompatibility. Chitosan was additionally responsible for the bactericidal effect against *E.coli* and *S.aureus*. The authors indicate this composition as a promising scaffold for skin regeneration [149]. Blends containing chitosan and collagen are also considered as a bioink for obtaining 3D structures [147]. A popular solution is also the addition of nanoparticles to such blends to improve antibacterial activity [150]. Combinations of chitosan with hyaluronic acid [143], gelatin [151,152], cellulose [153,154], and starch have also been researched.

## 8. The Comparison of Existing Knowledge in the Field of Chitosan Application in Cosmetics

**Figure 10:**
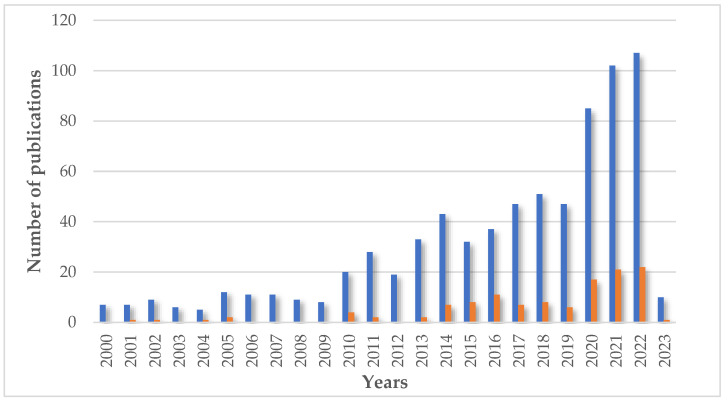
The number of publications in the years about chitosan in cosmetics (▪ search for words “chitosan” and “cosmetics”) and final products based on chitosan (▪ search for term “cosmetic based on chitosan”). Figure is based on data from Scopus database (in both cases, including title, abstract, and keywords; [https://www.scopus.com/search/form.uri?display=basic#basic; accessed on February 2023]).

Nowadays, chitosan is a very popular biopolymer in many fields. The results of a search in the scientific literature for the word “chitosan” indicate a significant increase in interest in this macromolecule, especially since the early 2000s. According to the Scopus database, at the beginning of the 21st century, the annual number of results (including title, abstract, and keywords) for this term was about 500, and in December 2022 (the access date), it is over 9500 for this year. The total search results number is approximately 95,839 papers. The main areas of chitosan interest are materials science, chemistry, biochemistry, and engineering. Cosmetic applications are not a very popular topic in the literature. Only 758 results of documents appear in title, abstract, and keyword searches for the words “chitosan” and “cosmetics” together. When the search takes only the title, even fewer results—29—are presented. Within these records, the main fields are chemistry, biochemistry, chemical engineering, and medicine. The cosmetics industry is very specific, and cosmetics companies do not publish research results very often (Figure 10).

The popularity of chitosan is the result of looking for sustainable solutions in the field of obtaining polymers and their processing and utilization, but above all, this biopolymer has a number of desirable properties and shows a wide range of activity. The multitude of chitosan applications is due to its polycationic character, which is unique in nature. The most significant properties regarding cosmetics are film-forming [10,155,156], antioxidant [10,34,106,113], and antimicrobial [106,124,157]. Due to the fact that cosmetics are mainly in contact with the skin and mucous membranes, the cosmetics industry can draw on many achievements of dermatology and regenerative medicine in the use of chitosan. Despite the beneficial properties of this biopolymer, there are several potential problems, mainly related to its source and production method. Most commercially available chitosan products are of marine origin and are obtained by a chemical process. This is not always a suitable source for cosmetic applications due to the risk of zoonotic diseases and also ethical and environmental issues related to biodiversity and the protection of endangered species [158]. Furthermore, in the industrial production of chitosan, it is extremely difficult to obtain a product with sufficient purity and uniform parameters. Scientists are working on alternative methods of production, such as enzymatic methods, including fermentation [32]. This biotechnological process is promising one of the new methods will be fully sustainable. This is probably a very good direction for the chemistry of cosmetics, where there is a strong trend for natural cosmetics [159,160,161]. Another future direction may be work on obtaining chitosan from alternative sources, including insects and fungi, in which case the problem of the seasonality of the obtained material is bypassed. Another consumer trend, including fully vegan cosmetic products, may shift the use of this biopolymer towards only chitosan of fungal origin. Additionally, a promising direction for chitosan in the cosmetic market is the increasing use of nanotechnologies, especially nanocapsules, for encapsulating active ingredients. Chitosan in this form not only protects the active ingredients from degradation, extending the shelf life of the cosmetic product, but also ensures a time-controlled release after application to the skin [162].

## 9. Conclusions

Chitosan and its derivatives are multifunctional ingredients in cosmetic formulations. Despite the small number of published research results in this field, chitosan is successfully used in various types of cosmetic forms (such as creams, foams, and gels) for various purposes (moisturizing, deodorizing, regenerating, and supporting the alleviation of specific skin ailments). In cosmetics, it acts as a rheology modifier, improves the stability of the formulation and allows limiting the use of preservatives. Proven antioxidant and antibacterial properties and penetration skin abilities allow it to be called cosmeceutical and not just a care ingredient. As the main limitation in the use of pure chitosan, the poor solubility in water at neutral pH is indicated; however, thanks to the presence of active groups in its structure, it is possible to easily carry out modifications that improve this condition. Its biocompatibility, biodegradability, and non-toxicity are also of great importance. All these factors make chitosan an almost ideal ingredient for cosmetic applications, but it is still too little widespread in this field compared to biomedicine, tissue engineering, or the food industry.

## Figures and Tables

**Table 1:** Minimal inhibitory concentration of chitosan for selected bacterial strains (including acne-related bacteria) [109,110].

Bacterial Strains	MIC of Chitosan (μg/mL)
*Vibrio cholerae*	60
*Pseudomonas aureginosa*	32/60
*Staphylococcus aureus*	16/80
*Staphylococcus epidermidis*	64
*Cutibacterium acnes*	512
*Streptococcus* sp.	60
*Salmonella* sp.	80
*Escherichia coli*	80
*Proteus vulgaris*	50

## Data Availability

No new data were created or analyzed in this study. Data sharing is not applicable to this article.
